# ZCCHC14 regulates proliferation and invasion of non–small cell lung cancer through the MAPK‐P38 signalling pathway

**DOI:** 10.1111/jcmm.16223

**Published:** 2020-12-20

**Authors:** Xiuying Shi, Xu Han, Yu Cao, Cheng Li, Yaming Cao

**Affiliations:** ^1^ Department of Immunology The College of Basic Medical Sciences China Medical University Shenyang China; ^2^ Department of Pathology The College of Basic Medical Sciences China Medical University Shenyang China; ^3^ Department of Breast Surgery The First Hospital of China Medical University Shenyang China

**Keywords:** invasion, lung cancer, P38 signalling pathway, proliferation, ZCCHC14

## Abstract

ZCCHC14 is a CCHC‐type zinc finger protein which is expressed in tissues in human and mouse. The function of ZCCHC14 in tumours remains unclear. In this research, we explored the expression, function and related molecular mechanisms of ZCCHC14 in human non–small cell lung cancer (NSCLC). Immunochemistry staining showed that ZCCHC14 was low‐expressed or absent in NSCLC tissues. In NSCLC patients, the low expression of ZCCHC14 in tumour tissues was significantly correlated with TNM stage, differentiation degree and adverse clinical outcome (*P* < .05). The proliferation and invasion ability of cancer cells transfected with ZCCHC14 CRISPR/Ca9 KO plasmids was significantly enhanced (*P* < .05). Immunoblotting analysis indicated that the expression of p‐P38, cyclinD1 and MMP7 were significantly up‐regulated after disabling ZCCHC14 (*P* < .05). We used MAPK‐P38 pathway inhibitor doramapimod (BIRB 796) to inhibit P38 signalling pathway activity and determined that the agent significantly disrupted the function of ZCCHC14 and hindered the proliferation and invasion of the tumour. The finding revealed that ZCCHC14 can regulate proliferation and invasion of NSCLC through the P38 pathway. ZCCHC14 plays a crucial regulatory role in the development of NSCLC and may become a zinc finger target for clinical treatment.

## 1 INTRODUCTION

ZCCHC14 is a CCHC‐type zinc finger protein of approximately 100 kDa size and possessing 14 CCHC domains. ZCCHC14 mRNA had been detected in almost all organs such as human brain, lung and liver. Studies have shown that CCHC‐type zinc finger proteins can bind DNA or RNA and play roles in regulating tumour progression by mediating protein interactions.^1^ Most CCHC‐type zinc finger proteins play biological functions closely related to changes of the MAPK signalling pathway^2^, ^3^, ^4^, ^5^ composed of P‐38, ERK1/2, ERK5 and JNK.^6^

It is generally acknowledged that smoking and alcohol are independent risk factors for lung cancer. Using meta‐analysis associated with whole genome analysis, researchers determined that the SNP of *ZCCHC14* may be caused by susceptibility of nicotine dependence (ND) and a reason for alcohol addiction.^7^ Smoking and alcohol intake in turn carry a higher risk of developing tumour. In addition, a Genome Wide Association Study (GWAS) indicated that the leading single‐nucleotide polymorphism in small vessel stroke is associated with mRNA expression of *ZCCHC14* in arterial tissues,^8^ indicating that *ZCCHC14* may play a role in cerebrovascular formation and management. ZCCHC14 might be associated with the progress of tumour metastasis, such as brain metastasis, although the function of ZCCHC14 in tumour has not been researched.

In our work, we investigated differences in the expression of ZCCHC14 in normal human lung tissue and NSCLC tissue and addressed the function of ZCCHC14 and its molecular mechanisms in the progression of NSCLC.

## 2 MATERIALS AND METHODS

### 2.1 Tissue samples

Paraffin‐embedded tissue samples were obtained from 104 NSCLC specimens (79 adenocarcinoma and 25 squamous cell carcinoma), 26 specimens of paired non‐tumour portions (>5 cm distance from the edge of the primary tumours) and 48 specimens of brain metastases. Follow‐up data were available for 46 cases. The TNM stages of tumours were revised by the Union for International Cancer Control (UICC).^9^, ^10^ All samples were obtained between 2012 and 2015 from surgical resection before patients received radiotherapy, chemotherapy or immunotherapy at the First Affiliated Hospital of China Medical University. This study was supervised by the Institutional Review Board of China Medical University. All participants provided informed consent.

### 2.2 Immunohistochemistry

Paraffin sections were dewaxed and antigen repaired. Immunohistochemistry staining using ZCCHC14 polyclonal antibody (ab‐150591, Abcam, USA) was performed in accordance with the kit instructions (KIT9720, MXB, China). The sections were sealed with Neutral balsam after dehydration. The grades of immunostaining were assessed as described.^10^

### 2.3 Immunofluorescence assay

Cells on coverslips were fixed with cold methanol for 10 minutes. They were incubated with anti‐ZCCHC14 antibody (ab‐150591, Abcam, USA) after blocking with 5% BSA. Immunofluorescence assay was performed using secondary antibodies conjugated to tetramethyl rhodamine isothiocyanate (TRITC) for protein staining and DAPI for nuclear staining. The staining was detected using a fluorescence microscope (Olympus IX51, Japan) and images captured with a camera (CoolPIX 5400; Nikon, Japan).

### 2.4 Immunoblotting analysis

Immunoblotting analysis was performed as described.^9^ 5% BSA and non‐fat milk were used as blocking reagent. The corresponding antibodies mainly included ZCCHC14 (ab‐150591, Abcam, USA), P42/44 (AM076, Beyotime, China), p‐P42/44 (AM071, Beyotime, China), cyclinD1 (AC853, Beyotime, China), MMP7 (D4H5; 3801, Cell Signaling, USA), MMP9 (D6O3H; 13 667, Cell Signaling, USA), GADPH (ab8245, Abcam, USA), JNK (AJ518; Beyotime, China), P‐JNK (AJ516, Beyotime, China), P38MAPK (AM065, Beyotime, China) and p‐P38MAPK (AM063, Beyotime, China),

### 2.5 Cell culture and transfection

The cell lines included human bronchial epithelial cell HBE, and lung cancer cell lines A549, H1299, H460, LK2 and H292 were purchased from the American Type Culture Collection (Manassas, VA, USA). Cells were continuously cultured in RPMI‐1640 which contained 10% foetal bovine serum (Gibco, AUS) at 37°C in an incubator with 5% CO_2_. The cells were seeded in a 6‐well plate before experiments. ZCCHC14 CRISPR/Ca9 KO(sc‐411686, Santa Cruz Biotechnology, USA) and Lipofectamine 3000 (Invitrogen, Thermo Fisher, USA) was used for transfection and cells were collected at 48 hours. DMSO was added for comparison. We purchased P38 pathway inhibitor doramapimod (BIRB 796) from Selleckchem (S1574, USA).

### 2.6 Colony formation assay

The colony forming ability of tumour cells was examined as described.^11^ In brief, cell suspensions with 3000 cells were blended evenly and incubated in medium for 10 days following placing in 6‐cm cell culture dishes. Haematoxylin was used to stain the cells, and colonies > 0.3 mm were counted. Experiments were repeated three times.

### 2.7 Transwell assay

Transwell assays were performed as described.^11^ Cell suspensions (100 µl containing 1 × 10^4^ cells) were placed in the upper chamber and were then incubated for an additional 24 hours. Cells appearing on the lower surface of the filter were stained with crystal violet and five random fields were counted using a high‐magnification microscope. Experiments were repeated three times.

### 2.8 Statistical analysis

SPSS statistical software package version 13.0 (SPSS Inc, Chicago, IL) was used for analysing date. All results were analysed using Pearson's chi‐square test. Experiments were repeated three times. The data were shown as mean ± standard deviation (SD), and significance level was set 5%.

## 3 RESULTS

### 3.1 Aberrant ZCCHC14 expression was significantly correlated with tumour progression and poor survival of patients

**FIGURE 1:**
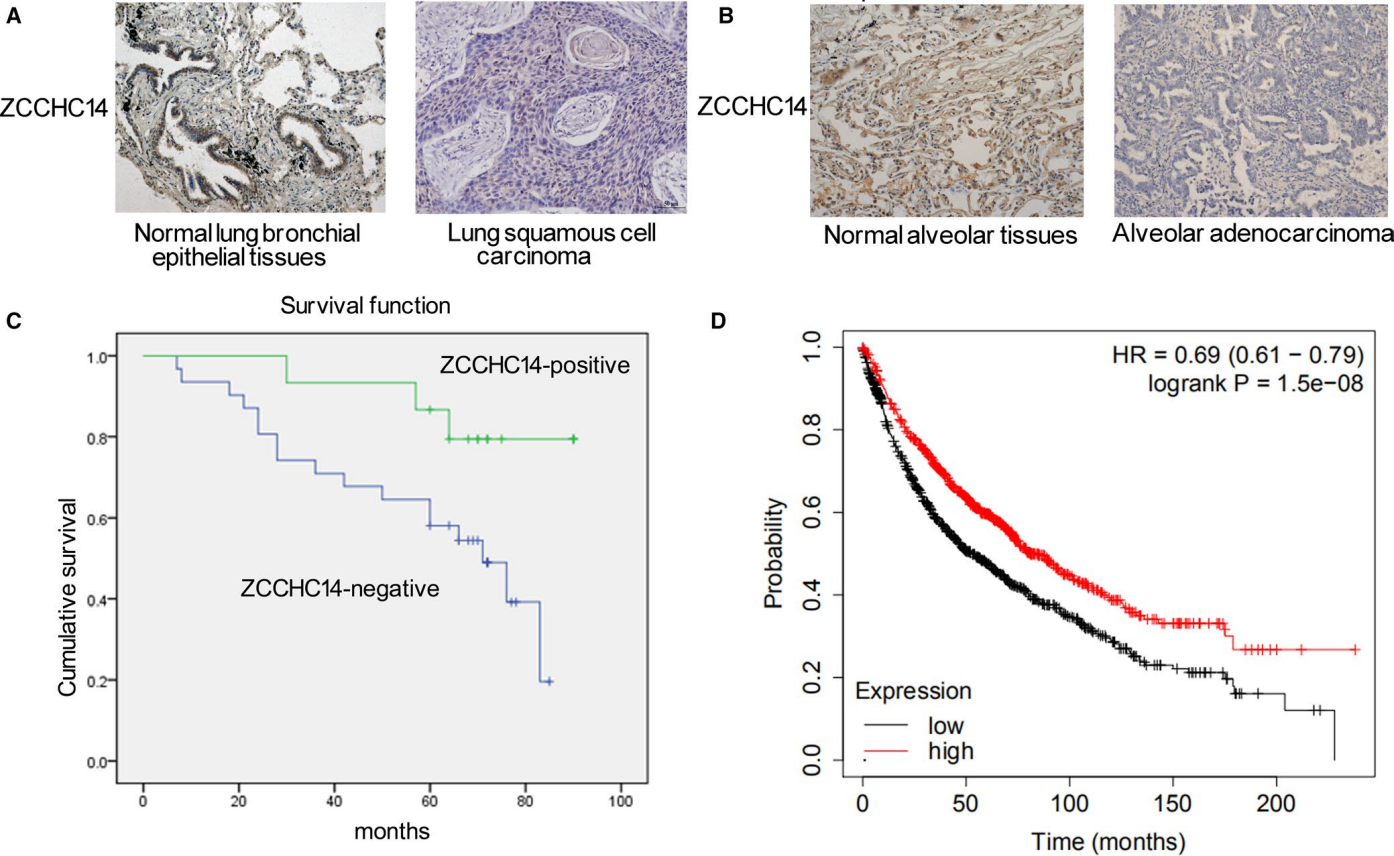
Immunohistochemical analysis of expression of ZCCHC14 in normal lung tissues and NSCLC tissues. A, Immunohistochemical experiments showed that ZCCHC14 was observed in normal lung bronchial epithelial tissues and weakened or absent in lung squamous cell carcinoma. Original magnification, ×200. B, expression of ZCCHC14 was normal in normal alveolar tissues and low or absent in adenocarcinoma. C, the survival time of patients with ZCCHC14 protein expression in NSCLC was analysed by the overall Kaplan‐Meier survival curve. D, Kaplan–Meier curves of ZCCHC14 mRNA expression in NSCLC as retrieved from the KM Plotter database

Immunochemistry staining was firstly performed to investigate the expression of ZCCHC14. It was found that ZCCHC14 was expressed in normal bronchial epithelial cells and was weaker or absent in lung squamous cancer cells (Figure 1A). Expression of ZCCHC14 was normal in alveolar cells and low or absent in adenocarcinoma cells of NSCLC patients (Figure 1B). The ZCCHC14 negative rate was 73% in lung cancer tissues (76/104) and 90% brain metastases tissues (43/48), and both differences were statistically significant (*P* < .05) (Table 1). The low expression of ZCCHC14 in cancer tissues was significantly correlated with poor versus good differentiation, TNM advanced stage (III + IV) and male gender (*P* < .05) (Table 1). The survival analysis revealed that the survival time of patients without ZCCHC14 expression in tumour tissues (60 ± 5 m) was significantly shorter than that of patients with ZCCHC14 expression (82 ± 4 m) (*P* < .05) (Figure 1C). To verify the expression and prognostic value of ZCCHC14, we retrieved the online database Kaplan‐Meier Plotter (KM Plotter) (http://kmplot.com/analysis/index.php).^12^ According to the KM Plotter database, patients with NSCLC with high expression of ZCCHC14 mRNA had longer survival probability than that of patients with low levels of ZCCHC14 mRNA expression (*P* < .001; Figure 1D).

**TABLE 1:** Relationship between ZCCHC14 expression and clinicopathological features in non–small cell lung cancer

Factors	Number of patients	ZCCHC14 expression		*P* value
		Negative	Positive	
Age, y				
<55	38	29 (76%)	9 (24%)	.572
≥55	66	47 (71%)	19 (29%)	
Gender				
Male	46	41 (89%)	5 (11%)	.001
Female	58	35 (60%)	23 (40%)	
Grade				
Well and moderate	61	38 (62%)	23 (38%)	.003
Poor	43	38 (88%)	5 (12%)	
TNM stage				
I and II	49	31 (63%)	18 (37%)	.033
III and IV	55	45 (82%)	10 (18%)	
Brain metastases				
No	104	76 (73%)	28 (27%)	.022
Yes	48	43 (90%)	5 (10%)	

Note

*P* values were obtained with the chi‐squared test.

### 3.2 Low expression of ZCCHC14 in lung cancer cells promoted proliferation and invasion of cancer cells in vitro

**FIGURE 2:**
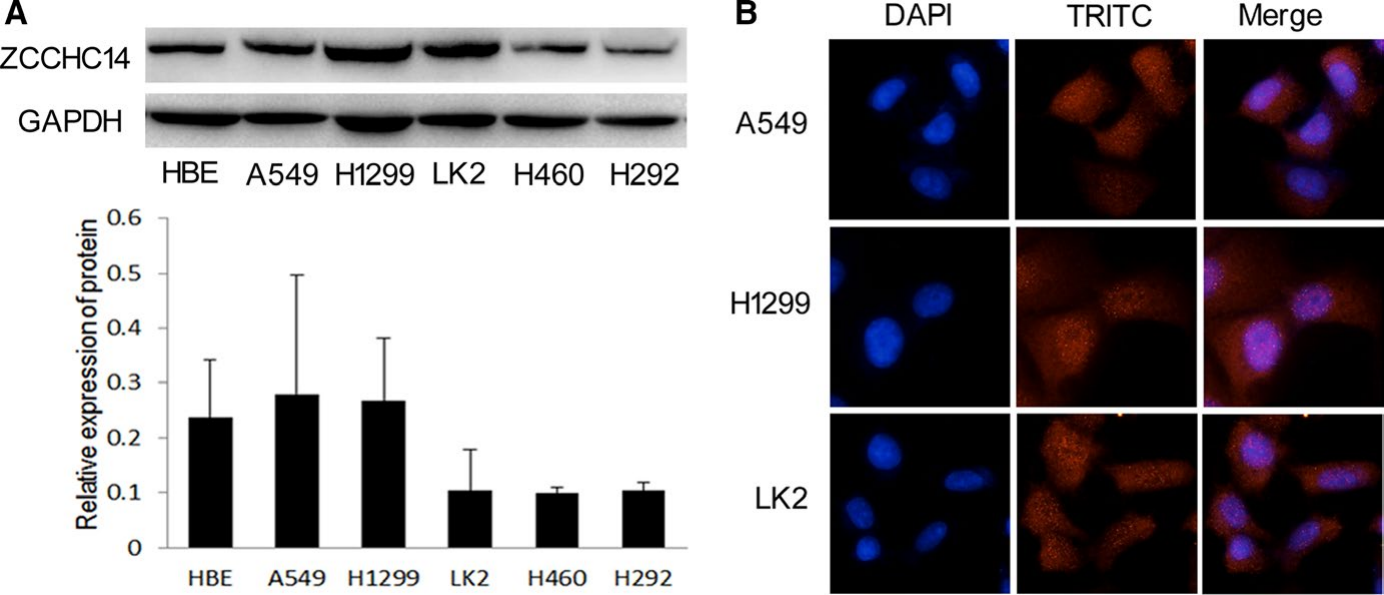
Expression and location of ZCCHC14 in cell lines in vitro. A, expression of ZCCHC14 was analysed by Western blot and visualized by ZCCHC14 antibody (Abcam). GAPDH (Abcam) was detected as a loading control. B, the location of ZCCHC14 in cancer cells was shown as red fluorescent dots counterstained with DAPI. Original magnification, ×400

**FIGURE 3:**
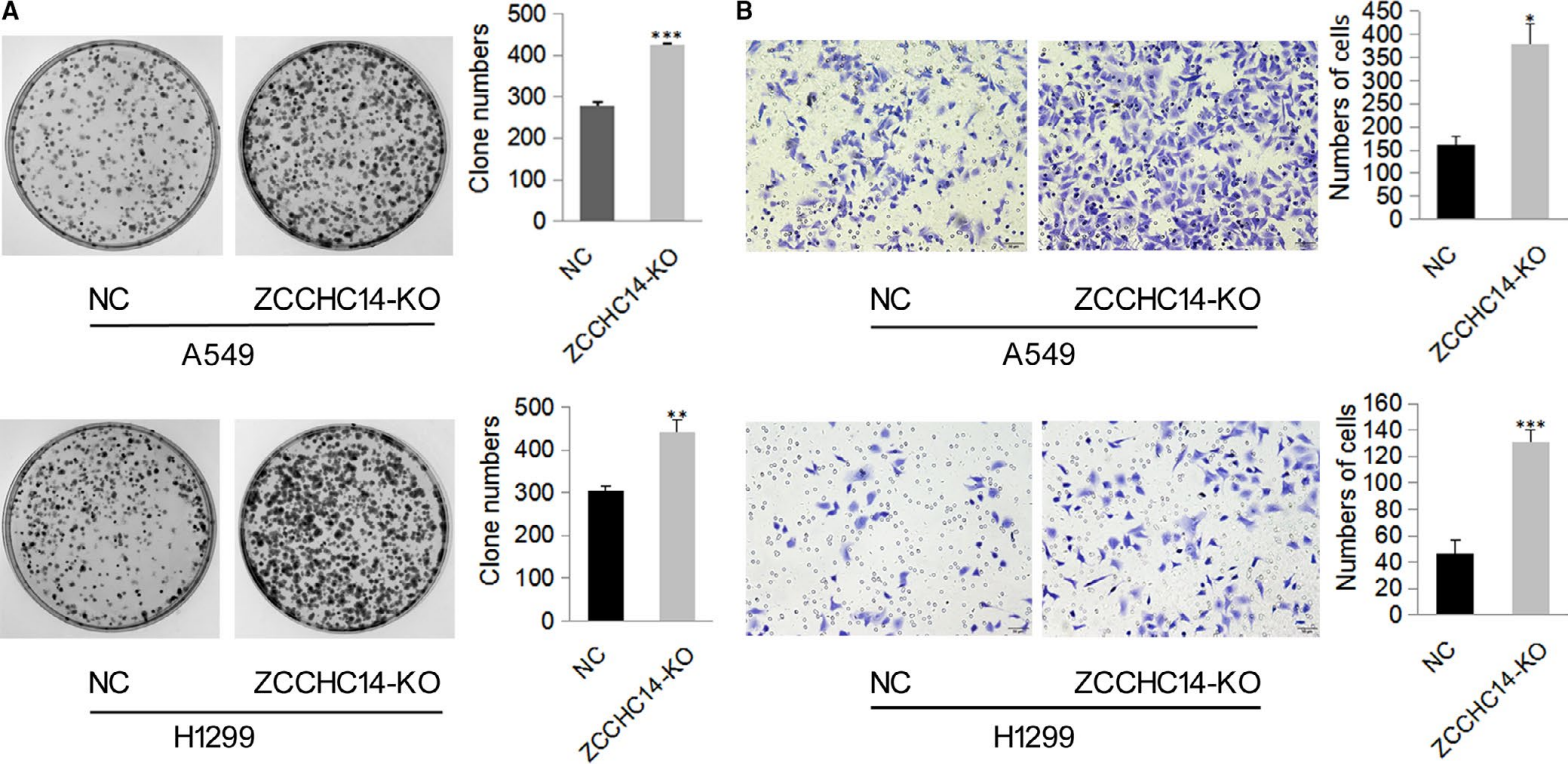
Knockdown of ZCCHC14 promotes A549 cell and H1299 cell proliferation and invasion. A, the cells (3000 cells/dish) were treated with an indicated dose of ZCCHC14 CRISPR/Ca9 KO plasmids for 24 hours and incubated in fresh medium for another 10 days. Typical pictures are shown in phase contrast mode. Proliferation ability is expressed as the number of colonies shown in the histogram. Data are depicted as mean ± SD. NC, negative control cells; ZCCHC‐KO, cells transfected with ZCCHC14 CRISPR/Ca9 KO plasmids; ns, no significance. *, *P* < .05; **, *P* < .01; ***, *P* < .001. B, Representative images of the invasion assays for H1299 and A549 cells (100,000 cells/well) transfected with ZCCHC14‐KO plasmids and control cells. The invasive cell number for each group is shown in the histogram. Original magnification, ×200

To study the function of ZCCHC14 in cancer cells, we conducted in vitro experiments. Firstly, the expression of ZCCHC14 was detected in HBE cells and lung cancer cells A549, H1299, LK2, H460 and H292. Immunoblotting analysis showed that ZCCHC14 was expressed in HBE and lung cancer cells and was under expressed to varying degrees in LK2, H460 and H292 cells (Figure 2A). Immunofluorescence staining experiments showed that ZCCHC14 was primarily localized on the nucleus of A549 and H1299 cells, and nucleus and cytoplasm of LK2 cells (Figure 2B). A549 and H1299 cells with appropriate expression of ZCCHC14 were selected for further experiments and transfected with ZCCHC14 CRISPR/Ca9 KO (ZCCHC‐KO) plasmids. Colony formation experiments showed that down‐regulation of ZCCHC14 significantly promoted the proliferation of cancer cells (*P* < .05) (Figure 3A). The results of transwell assays showed that the invasion ability of cancer cells was significantly increased after down‐regulating the expression of ZCCHC14 (*P* < .05) (Figure 3B).

### 3.3 ZCCHC14 regulates molecules downstream of the P38 pathway

**FIGURE 4:**
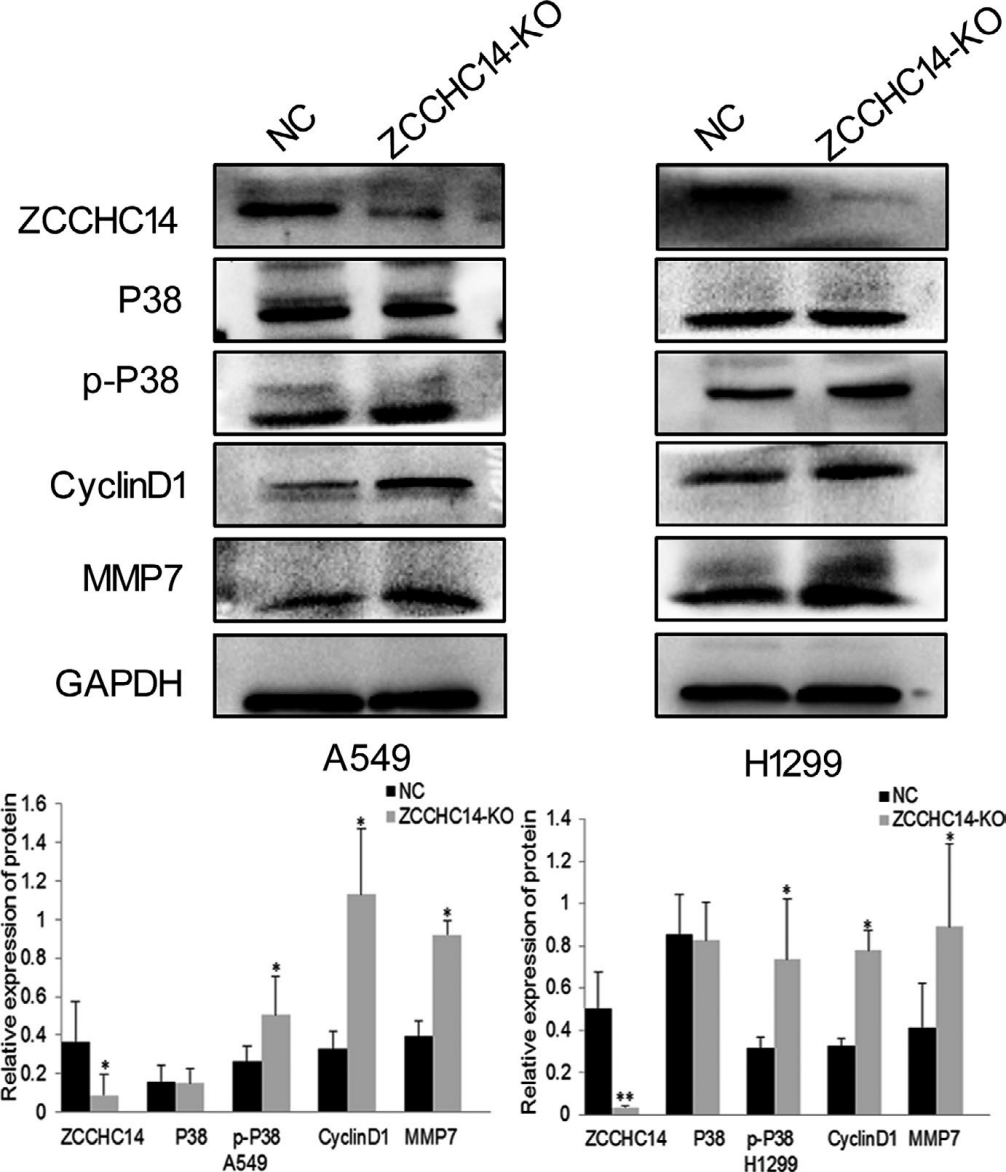
Expression of P38 signalling pathway proteins and downstream molecules after transfection of ZCCHC14‐KO plasmids into A549 and H1299 cells. Treated group transfected with ZCCHC14‐KO plasmids into cancer cells and their control cells were probed with antibodies against ZCCHC14, P38, p‐P38, cyclinD1 and MMP‐7. GADPH was used as a loading control. Data of P38 signalling pathway proteins and downstream molecules are shown as bar graphs depict mean ± SD Experiments were repeated three times

We confirmed whether ZCCHC14 regulates the MAPK signalling pathway and the regulatory mechanisms of the subfamily. Immunoblotting analysis showed that the relative expression of p‐P38 significantly increased and P38 expression was not significantly regulated after transfecting ZCCHC14‐KO plasmids in A549 and H1299 cells (Figure 4). After ZCCHC14 was knocked out from A549 and H1299 cells, the downstream signalling molecules of the P38 molecular pathway were significantly increased, including cyclinD1 and MMP7 (Figure 4).

### 3.4 Addition of a P38 pathway inhibitor abolished the effect of ZCCHC14 to suppress cancer cell proliferation and invasion

**FIGURE 5:**
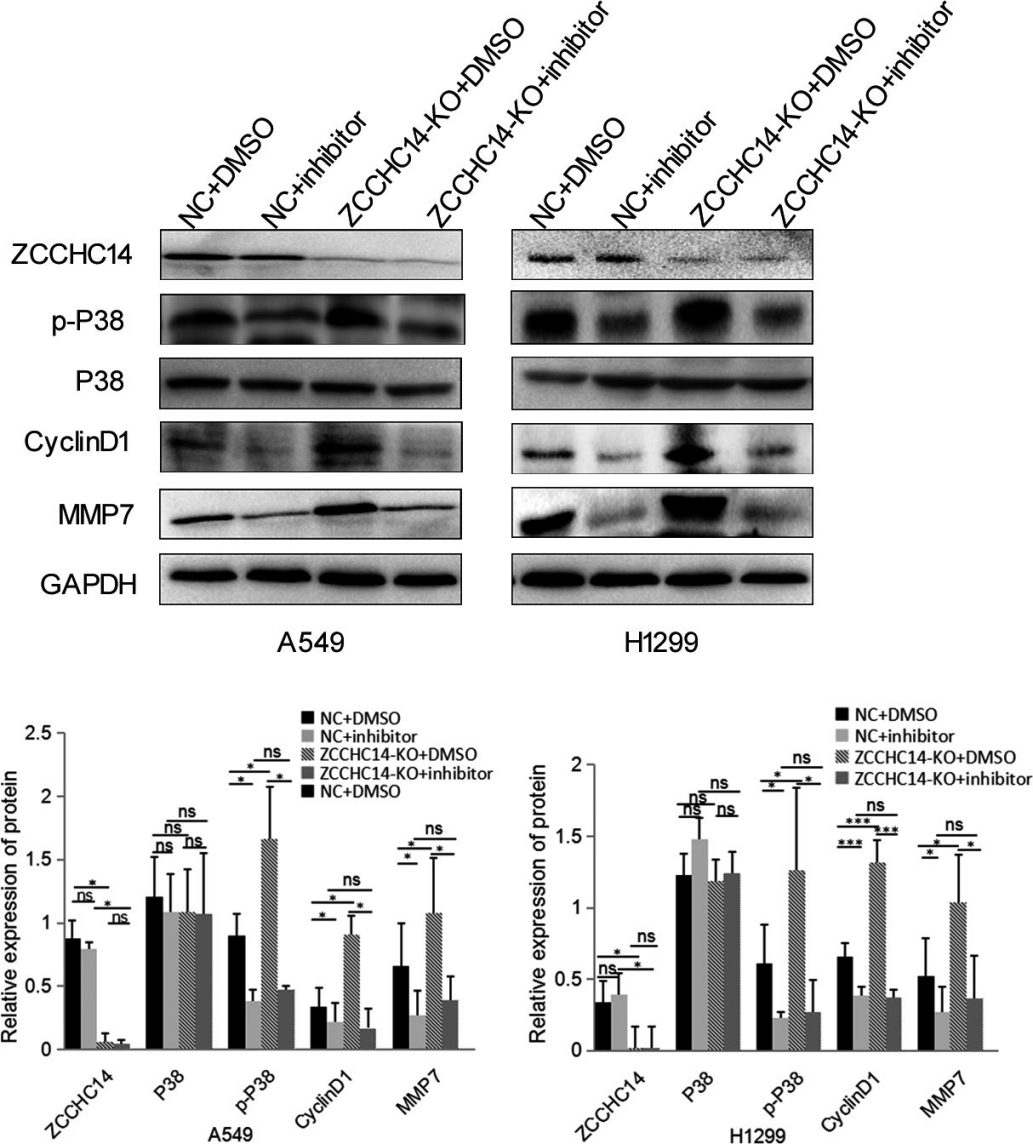
ZCCHC14 regulates P38 signalling pathway proteins and downstream molecules in vitro. Immunoblotting analysis was used to determine expression of ZCCHC14, P38, p‐P38, cyclinD1 and MMP7 after cells were transfected with a negative control plasmid (NC), ZCCCHC14‐KO plasmids, and/or P38 pathway inhibitor doramapimod. Data of P38 signalling pathway proteins and downstream molecules are shown as bar graphs depicting mean ± SD. Experiments were repeated three times

**FIGURE 6:**
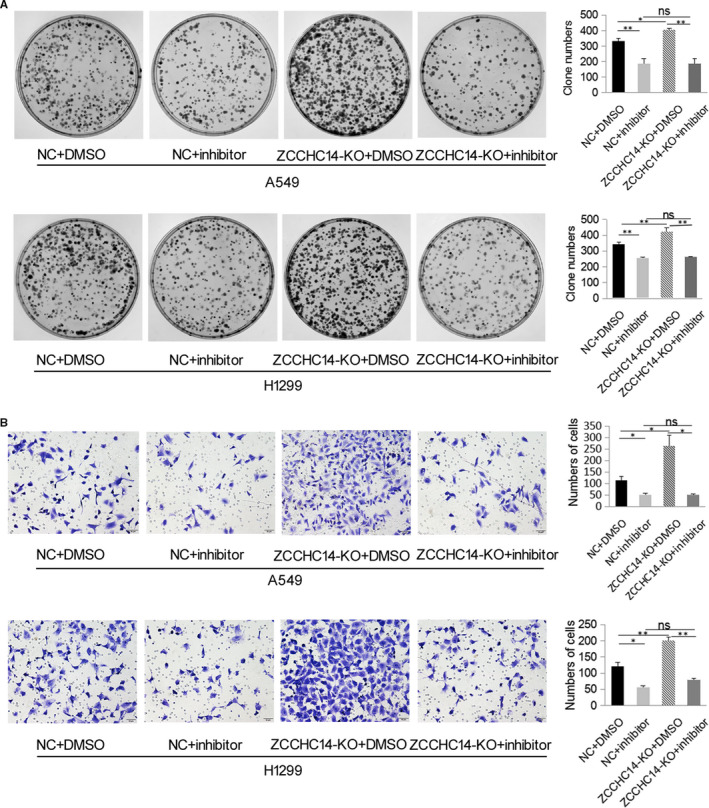
Addition of P38 pathway inhibitor abolished the effect of ZCCHC14 to suppress cancer cell proliferation and invasion in vitro. A, A549 and H1299 cells transfected with a negative control plasmid (NC), ZCCCHC14‐KO plasmids, and/or P38 pathway inhibitor doramapimod were then subjected to colony formation assays. Proliferation ability is expressed as the number of colonies shown in the histogram. Experiments were repeated three times. B, cells were treated as described in *A* and then subjected to transwell assays. The invasive cell number for each group is shown in the histogram. Original magnification, ×200

To determine whether ZCCHC14 plays a role in lung cancer cells through the P38 signalling pathway, we added the P38 pathway inhibitor doramapimod (BIRB 796). Immunoblotting analysis showed that p‐P38 expression significantly decreased after addition of doramapimod compared to cancer cells in the NC group (*P* < .05) (Figure 5). Colony formation assays showed that the addition of the inhibitor significantly reduced the proliferation ability of A549 and H1299 cells compared with the NC group (*P* < .05) (Figure 6A). Transwell assays showed that doramapimod significantly reduced the invasion of A549 and H1299 cells in the NC group (Figure 6B). Immunoblotting analysis showed that p‐P38 decreased significantly after the addition of inhibitor doramapimod with ZCCHC14‐KO plasmids compared with addition of ZCCHC14‐KO plasmids only (*P* < .05) (Figure 5), indicating that the function of ZCCHC14‐KO plasmids to promote P38 pathway activity was abolished by the inhibitor. Colony formation and transwell assays also showed that doramapimod inhibited the proliferation and invasion of cancer cells transfected with ZCCHC14‐KO plasmids (Figure 6A and B). Next, we compared the addition of doramapimod with ZCCHC14‐KO plasmids versus the addition of inhibitor alone. The p‐P38 expression in both groups was relatively low and did not significantly change (Figure 5), indicating that doramapimod inhibited the function of cancer cells transfected with ZCCHC14‐KO plasmids. Colony formation and transwell assays showed that the addition of doramapimod significantly reduced the proliferation and invasion of A549 and H1299 cells transfected with ZCCHC14‐KO plasmids (Figure 6A and B).

## 4 DISCUSSION

Most lung cancer patients with low survival rates are found in advanced stages.^13^ A clinical study revealed that brain metastasis is a crucial reason for disease progression and treatment failure of advanced NSCLC, and patients with brain metastases generally have a poor prognosis.^14^ Considering the incidence of NSCLC with brain metastases, and that the control of brain metastases is an important therapeutic target for metastatic NSCLC, in this study we included samples from patients with lung cancer and brain metastases.

It is important to find molecular changes which can help to distinguish lung cancer versus non–lung cancer patients and it will benefit the development of clinically targeted therapies often involved in biological functions. The MAPK signalling pathway participates in multifarious physiological and pathological processes^15^ and plays dual roles in different cell types and conditions.^6^ Studies have shown that the MAPK signal pathway plays a crucial part in preventing the occurrence and progression of cancer.^16^

Recent studies showed that ZCCHC14, a CCHC‐type zinc finger protein, is associated with nicotine dependence, alcohol addiction and small blood vessel stroke^7^, ^8^ and that ZCCHC14 in plasma has a positive effect on the prognosis of patients with cerebral haemorrhage.^17^ Otherwise, the role of ZCCHC14 in tumour tissues has never been researched. Our findings revealed that ZCCHC14 was expressed in normal bronchial epithelial cells and alveolar cells. In contrast to the expression of ZCCHC14 in normal lung tissues, the expression of ZCCHC14 in NSCLC cells was weakened and was absent in some patients. Our present work showed that ZCCHC14 is localized on the nucleus and cytoplasm. The mechanism of abnormal expression of ZCCHC14 in tumour tissues is unclear. Abnormal expression of ZCCHC14 in cancer tissue also suggests that it may have a different impact on cancer. Further analysis demonstrated that the expression of ZCCHC14 in NSCLC was significantly correlated with cancer development factors and clinical prognosis, indicating that it might represent a new molecular change in NSCLC. In particular, the expression of ZCCHC14 in patients with lung cancer and brain metastasis was different than in patients without brain metastasis, indicating that ZCCHC14 may be more relevant to the former condition. Using the KM Plotter database, we found that patients with high expression of ZCCHC14 mRNA had a longer survival time than those with low expression, which indirectly verified our experimental results.

Studies have shown that CCHC‐type zinc finger proteins play biological functions in tumour tissues via regulating the MAPK signalling pathway^2^, ^3^, ^4^, ^5^ composed of ERK1/2, P38, ERK5 and JNK.^18^ While ZCCHC14 is a protein containing multiple zinc finger motifs, there are no studies on its possible involvement in the signalling pathway. In this study, we firstly confirmed whether ZCCHC14 modulates the MAPK pathway and the regulatory mechanism of the subfamily. We found that ZCCHC14 inhibits the P38 pathway in lung tumour cells including A549 and H1299 cells, thereby inhibiting the proliferation and invasion of tumour cells. After the expression of ZCCHC14 was weakened, tumour proliferation and invasion ability were significantly increased and the phosphorylation level of the P38 signalling pathway was also significantly elevated. The introduction of the P38 inhibitor doramapimod further confirmed that ZCCHC14 regulates the biological function of cancer cells and phosphorylation of P38 through the P38 signalling pathway. However, the function of ZCCHC14 and its pathogenesis in lung cancer needs to be further analysed. These results only showed that the expression of ZCCHC14 has an antagonistic effect on the effect of the MAPK signalling pathway. In previous studies, HDAC1 was indirectly involved in the epigenetic regulation of P38 MAPK, which drives lung cancer progression.^15^ As an HDAC1 inhibitor, FK228 (depsipeptide) can act on non‐histone targets to enhance the anti‐tumour effect of TKI erotinib (for the treatment of NSCLC) by inhibiting the MAPK pathway18.^19^ Therefore, the pharmacological agents with combination of ZCCHC14, FK228 and erlotinib may be more effective in the treatment of NSCLC patients, and these may be more valuable in future clinical trials.

The proposed functions of ZCCHC14 include binding to nucleic acids, phosphatidylinositol, proteins and zinc ions. ZCCHC14 exerts an antagonistic effect through which function in lung cancer is unknown. Further study might determine if binding to phosphatidylinositol is involved, because the phosphatidylinositol receptor participates in the activation of P38 signalling pathway. The mechanism of how ZCCHC14 participates in the antagonistic action needs further study and may provide insights into molecular targeted therapies for lung cancer.

## CONFLICT OF INTEREST

The authors confirm that there are no conflicts of interest.

## AUTHOR CONTRIBUTIONS

**Xiuying Shi:** Conceptualization (lead); Data curation (lead); Formal analysis (equal); Methodology (equal); Writing‐original draft (lead). **Xu Han:** Methodology (equal); Supervision (supporting); Writing‐review & editing (supporting). **Yu Cao:** Funding acquisition (equal); Writing‐review & editing (supporting). **Cheng Li:** Software (equal); Supervision (supporting); Writing‐review & editing (equal). **Yaming Cao:** Conceptualization (equal); Funding acquisition (lead); Project administration (lead); Supervision (lead); Writing‐review & editing (lead).

## Data Availability

All date generated or used during the study are available from the corresponding author by request.
